# Supplementary material for: Targeting of apoptosis gene loci by reprogramming factors leads to selective eradication of leukemia cells
Source: Nat Commun. 2019 Dec 6;10:5594. doi: 10.1038/s41467-019-13411-y (PMC6898631; doi:10.1038/s41467-019-13411-y)
Supplement: Supplementary file 1 — Supplementary Information [file 41467_2019_13411_MOESM1_ESM.pdf]

Supplementary Information

Targeting of apoptosis gene loci by reprogramming factors leads to selective eradication of leukemia cells

Wang *et al.*

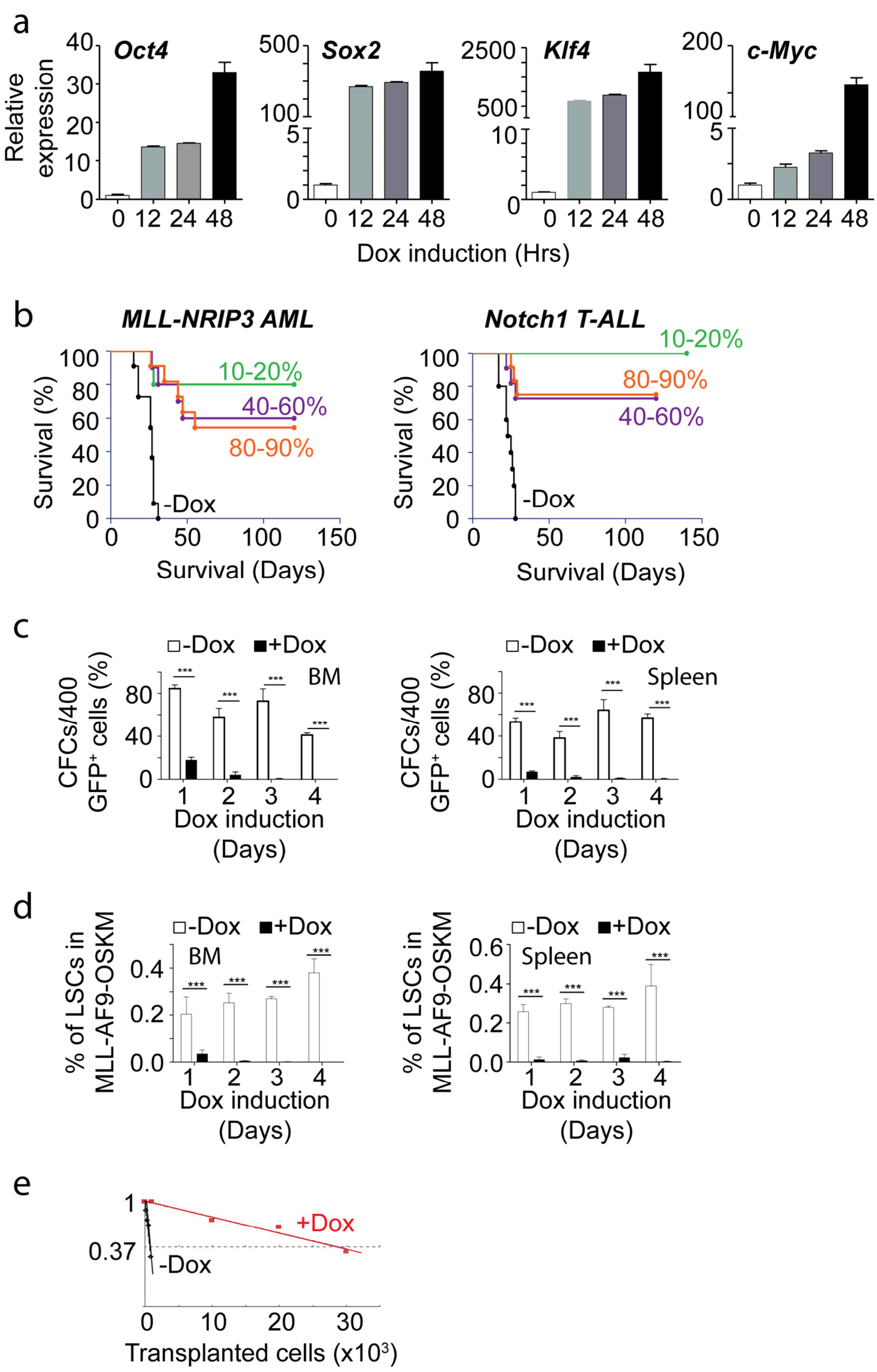

Supplementary Figure 1. OSKM selectively kill leukemia cells. (a) Gene expression of exogenous factors after Dox treatment. qRT-PCR analysis of the mRNA levels of OSKM genes in Dox-induced leukemia cells (n=3). One-way ANOVA. Error bars show SD. (b) Leukemia cell eradication in MLL-NRIP3-induced AML and Notch1-induced T-ALL. We established the MLL-NRIP3-induced AML and Notch1-induced T-ALL model using a similar strategy as shown in Figure 1a. In total,

2×10^5 AML or T-ALL cells were injected into sub-lethally irradiated (4.5 Gy) mouse recipients, and Dox was added to their drinking water at different time points. The data show the survival curves of the AML and T-ALL models (n=8, 2 independent experiments). (c) The bar charts show the colony forming ability of leukemia cells (GFP^+) that were isolated from Dox-induced bone marrow and spleen. Leukemia cells were treated with Dox for 24 hr, 48 hr, and 72 hr *in vivo*, and then GFP^+ cells were sorted from the bone marrow and spleen for colony assay (n=10, 2 independent experiments). ***$p < 0.001$, two-tailed Student's t test. Error bars show SEM. (d) The bar charts show the percentages of LSCs in GFP^+ leukemia cells after Dox treatment at different time points (n=8, 3 independent experiments). ***$p < 0.001$, two-tailed Student's t test. Error bars show SEM. (e) Limiting dilution assay. MLL-AF9-OSKM AML cells isolated from Dox treated (48 hr) or non-treated spleen were transplanted into sub-lethally irradiated (6 Gy) recipient mice. Leukemia-initiating cell frequencies and p-values were calculated by L-calc software.

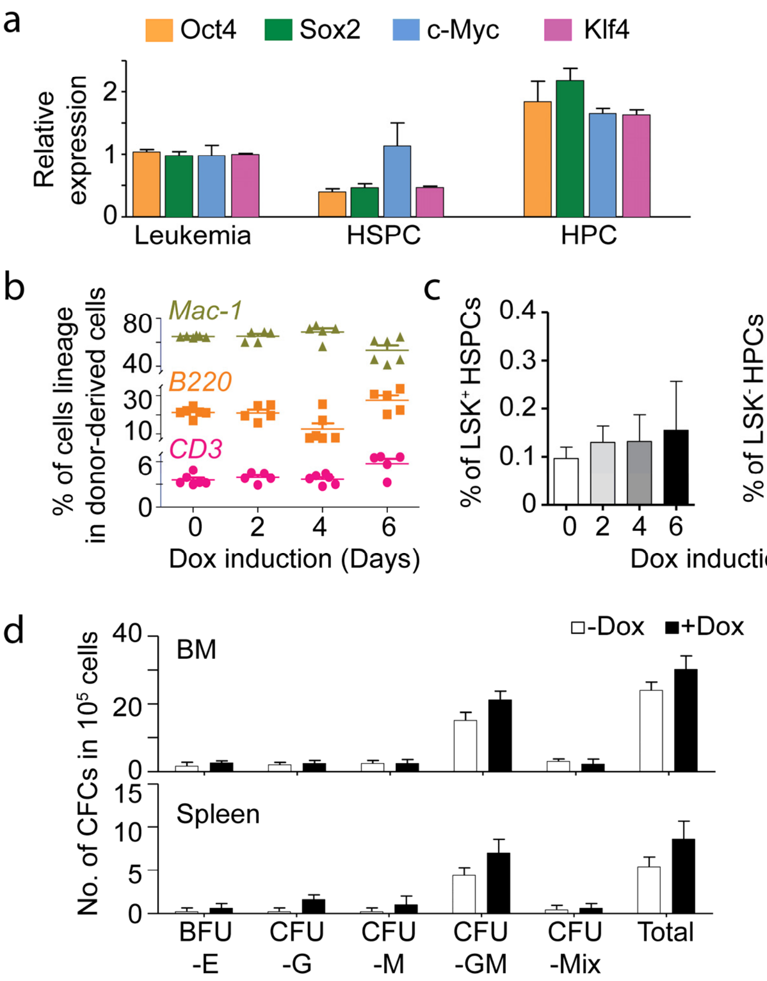

e

Cell types	Cytokines	Efficiency	Average efficiency
**HPCs** (lin^- $cKit^+$ $Sca\text{-}1^-$)	T,FL,3,6	$3320/10^5$ ~ $4200/10^5$	$3750/10^5$
**HSPCs** (lin^- $cKit^+$ $Sca\text{-}1^+$)	T,FL,3,6	$930/10^5$ ~ $1360/10^5$	$1200/10^5$
**Granulocytes** ($Gr\text{-}1^+$ $Mac\text{-}1^+$)	GM,G	$46/10^5$ ~ $67/10^5$	$56/10^5$
**Leukemia cells** (GFP^+)	S,3,6	$0/10^5$ ~ $6/10^5$	$0.6/10^5$
**Leukemia stem cells** (GFP^+ lin^- $cKit^+$ $Sca\text{-}1^-$ $IL\text{-}7R^-$)	S,3,6	–	–
**$Gr\text{-}1^-$ $Mac\text{-}1^+$ Leukemia cells** (GFP^+ $Gr\text{-}1^-$ $Mac\text{-}1^+$)	S,3,6	$0/10^5$ ~ $3/10^5$	$0.25/10^5$

Supplementary Figure 2. OSKM have minimal effect on normal hematopoietic cells. (a) Relative gene expression of ectopic *Oct4*, *Sox2*, *c-Myc* and *Klf4* in leukemia cells, HSPCs and HPCs after 48 hr of Dox induction (n=9, 3 independent experiments). One-way ANOVA. (b) Percentage of donor-derived mature cells (myeloid, T, and B cells) in the bone marrow after Dox treatment (n=10, 2

independent experiments). (c) Percentage of donor-derived LKS$^+$ HSPCs and LKS$^-$ HPCs in the bone marrow after Dox treatment (n=8, 2 independent experiments). One-way ANOVA. (d) CFC assay of normal hematopoietic cells from the bone marrow (left panel) and spleen (right panel) after 7 days of OSKM induction (n=5, 3 independent experiments). Two-tailed Student's t test. (e) The reprogramming efficiencies of different cell types were determined by counting AP$^+$ iPSC colonies. T, TPO; FL, Flt3-L; GM, GM-CSF; G, G-CSF; 3, IL-3; 6, IL-6. All error bars indicate SEM.

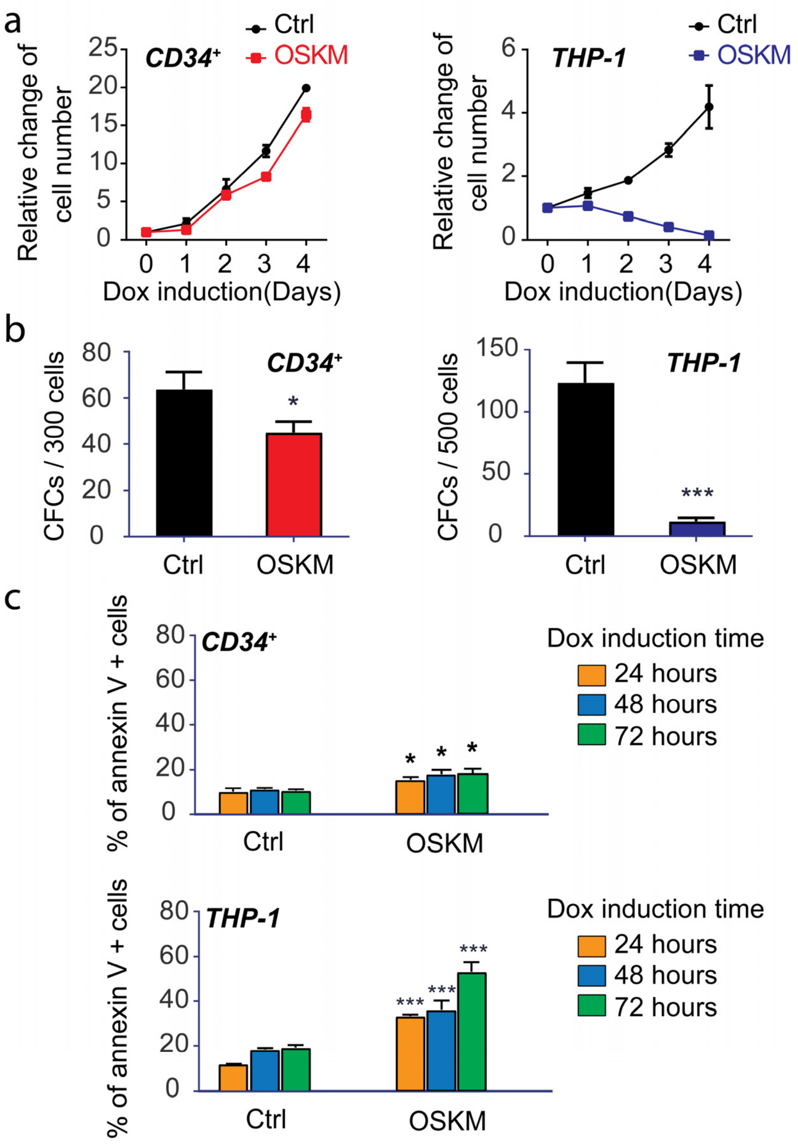

Supplementary Figure 3. OSKM selectively kill human leukemia cells. (a) *In vitro* liquid culture assays for human cord blood $CD34^+$ cells and THP-1 cells after OSKM ectopic overexpression. The cell number at day 0 was normalized to 1. The relative cell number at different time points is shown. 3 independent experiments. (b) Colony formation of human cord blood $CD34^+$ cells and THP-1 cells 48 hr after OSKM ectopic overexpression. 3 independent experiments. *p < 0.05, ***p < 0.001, two-tailed Student's t test. (c) Apoptotic analysis of human cord blood $CD34^+$ cells and THP-1 cells 24, 48, and 72 hr after OSKM ectopic overexpression. 3 independent experiments. *p < 0.05, ***p < 0.001, two-tailed Student's t test. All error bars show SEM.

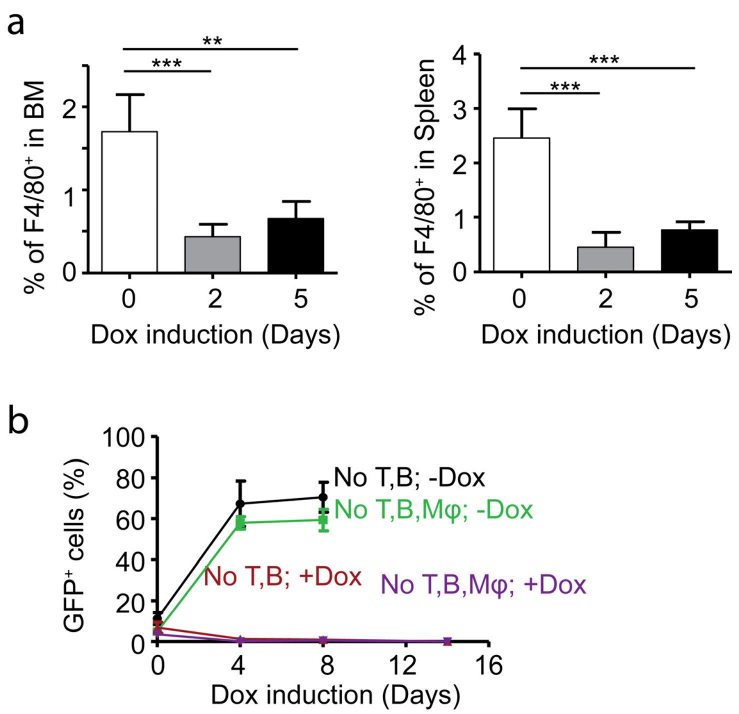

Supplementary Figure 4. Macrophage depletion assay. (a) Clearance efficiency of macrophages *in vivo*. Histograms show the percentages of macrophages in the bone marrow and spleen after 2 and 5 days of clodronate liposome (CL) treatment. **$p < 0.01$, ***$p < 0.001$, one-way ANOVA. Error bars show SD. (b) Changes of leukemia cells (GFP^{+}) in peripheral blood at different time points after Dox induction with different treatments (n=6-8, 2 independent experiments). Error bars show SEM.

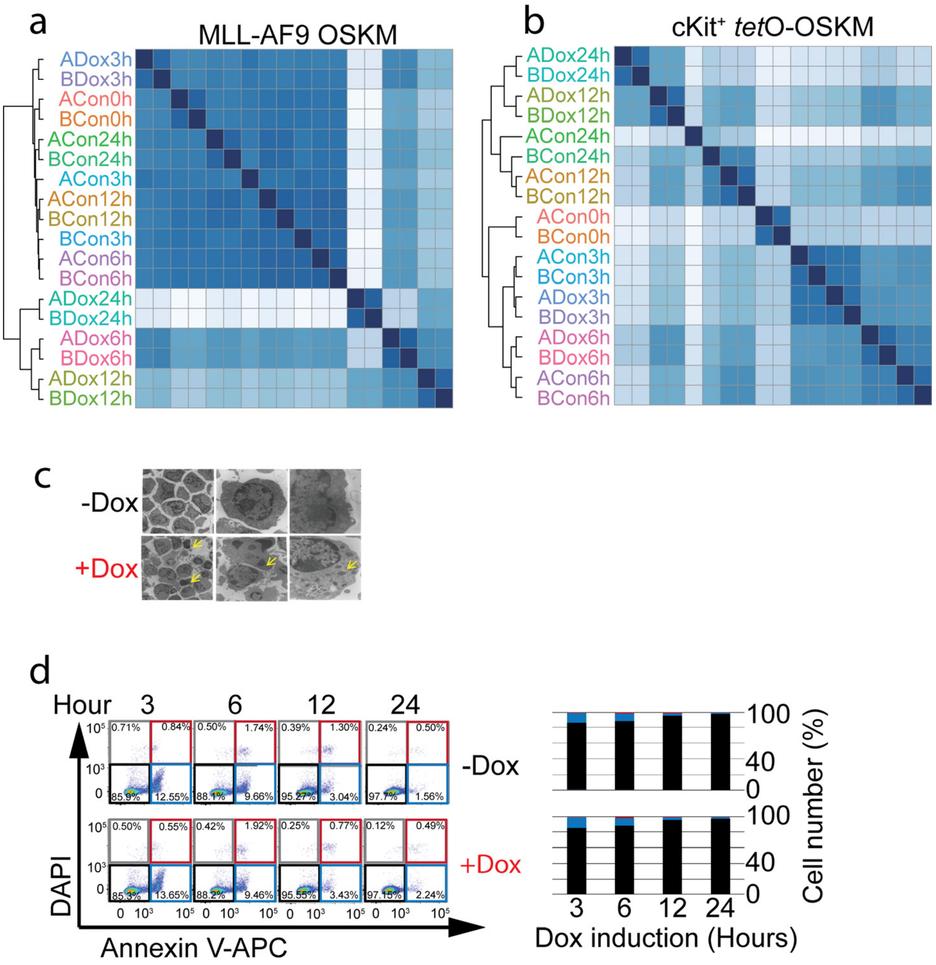

Supplementary Figure 5. Apoptosis is a primary cellular mechanism to eradicate leukemia. (a) RNA-seq cluster analysis of MLL-AF9-OSKM. The heatmap shows the sample distance of all MLL-AF9-OSKM samples. (b) RNA-seq cluster analysis of cKit$^+$ tetO-OSKM. The heatmap shows the sample distance of all *tet*O-OSKM cKit$^+$ samples. (c) Electron microscopy of leukemia mouse spleen tissue after 4 days of Dox induction. The image shows the following features: dilation of the mitochondria and karyolysis and pyknosis of leukemia cells in the spleen. Yellow arrow: apoptotic body. (d) Apoptosis of *tet*O-OSKM cKit$^+$ cells during 24 hr culture with or without Dox.

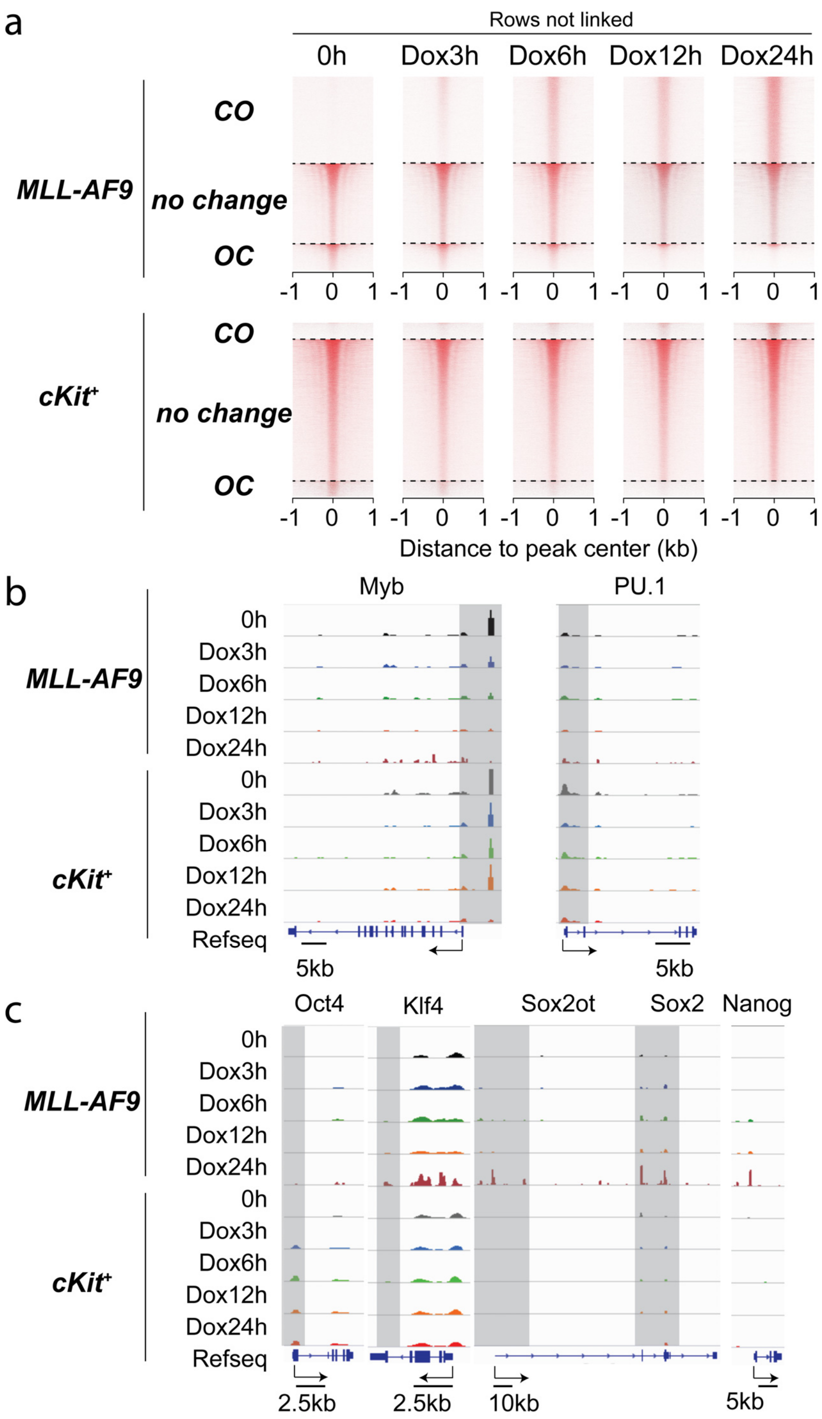

Supplementary Figure 6. Selected genomic view of OC/OC loci. (a) Heatmap of ATAC-seq tags for MLL-AF9-OSKM and *tet*O-OSKM cKit^{+} cells after Dox induction at different time points. The tags were centered around a 2-kb window relative to the midpoint of peak calls. Each row in the heatmap represents one chromatin open region (peak). The plot was descendingly sorted according to peak value. Therefore, the same row may not contain the same chromatin open region

among these different time points. (b) Integrative Genomics Viewer (IGV) view of *Myb* and *Pu.1* ATAC-seq data, CO/OC loci are marked with a gray box. In MLL-AF9-OSKM cells, *Myb* was closed after 3 hours. In *tet*O-OSKM cKit$^+$ cells, *Myb* was closed after 12 hours while *Pu.1* was closed after 3 hours. (c) IGV view of *Oct4*, *Klf4*, *Sox2*, *Sox2ot* and *Nanog* ATAC-seq data, CO/OC loci are marked with a gray box. In MLL-AF9-OSKM cells, *Klf4*, *Sox2* and *Nanog* were opened after 24 hours. In *tet*O-OSKM cKit$^+$ cells, *Oct4* was opened after 3 hours.

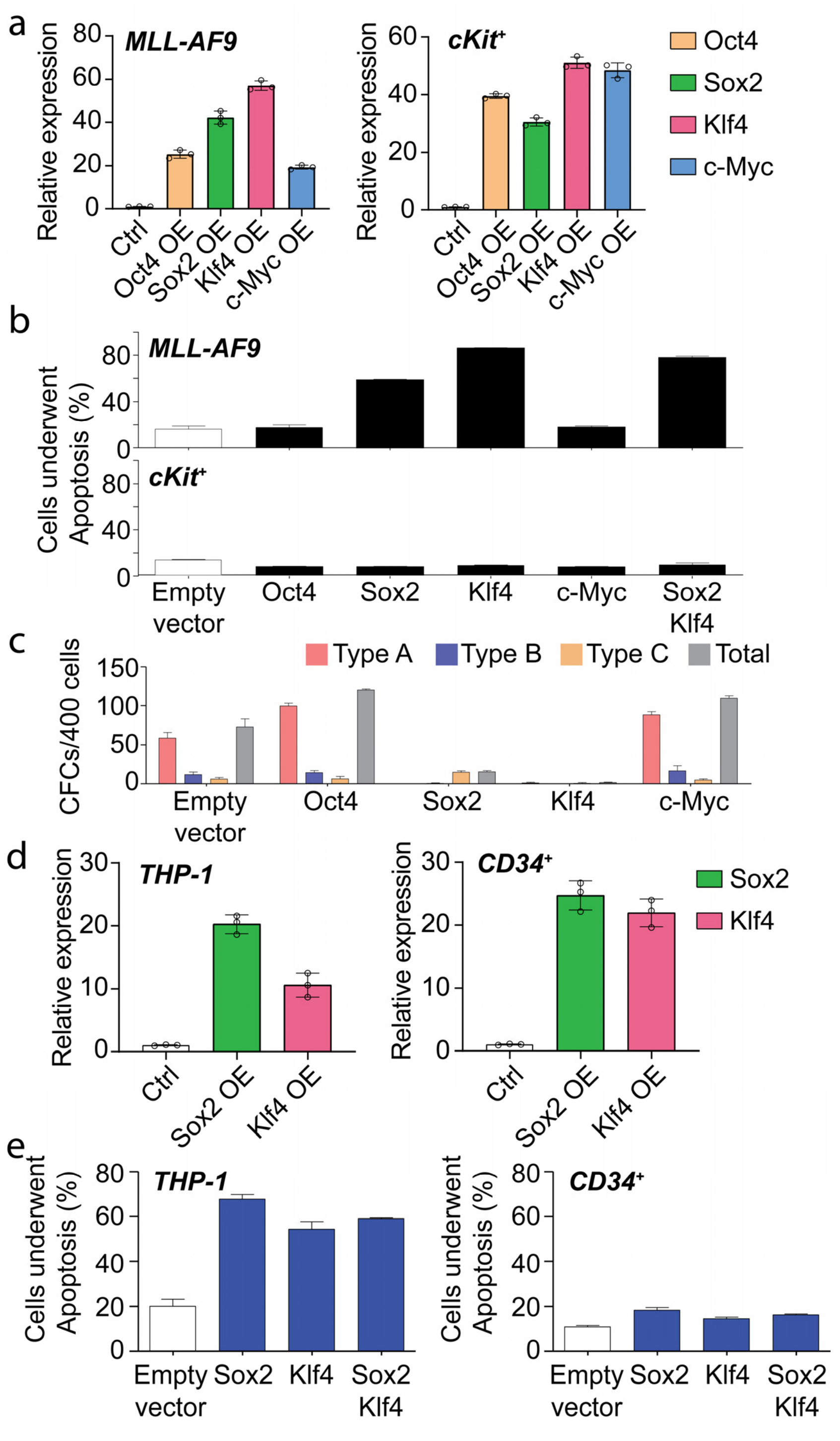

Supplementary Figure 7. Comparisons of the roles of individual OSKM factors. (a) qRT-PCR analysis of the mRNA levels of individual OSKM genes after 48 hr of lentivirus infection in AML cells and cKit$^+$ cells (n=3). Error bars show SD. (b) Apoptosis rate of AML cells and cKit$^+$ bone marrow cells after OSKM ectopic overexpression. 2 independent experiments. Error bars show SEM. (c) Colony assays of AML cells at 48 hr after OSKM ectopic overexpression (n=4, 2 independent

experiments). Error bars show SEM. (d) qRT-PCR analysis of the mRNA levels of *Sox2* and *Klf4* genes after 48 hr of lentivirus infection in THP-1 cells and CB $CD34^+$ cells (n=3). Error bars show SD. (e) Apoptosis of THP-1 cells and normal CB $CD34^+$ cells after *Sox2* and *Klf4* overexpression. 3 independent experiments. Error bars show SEM.

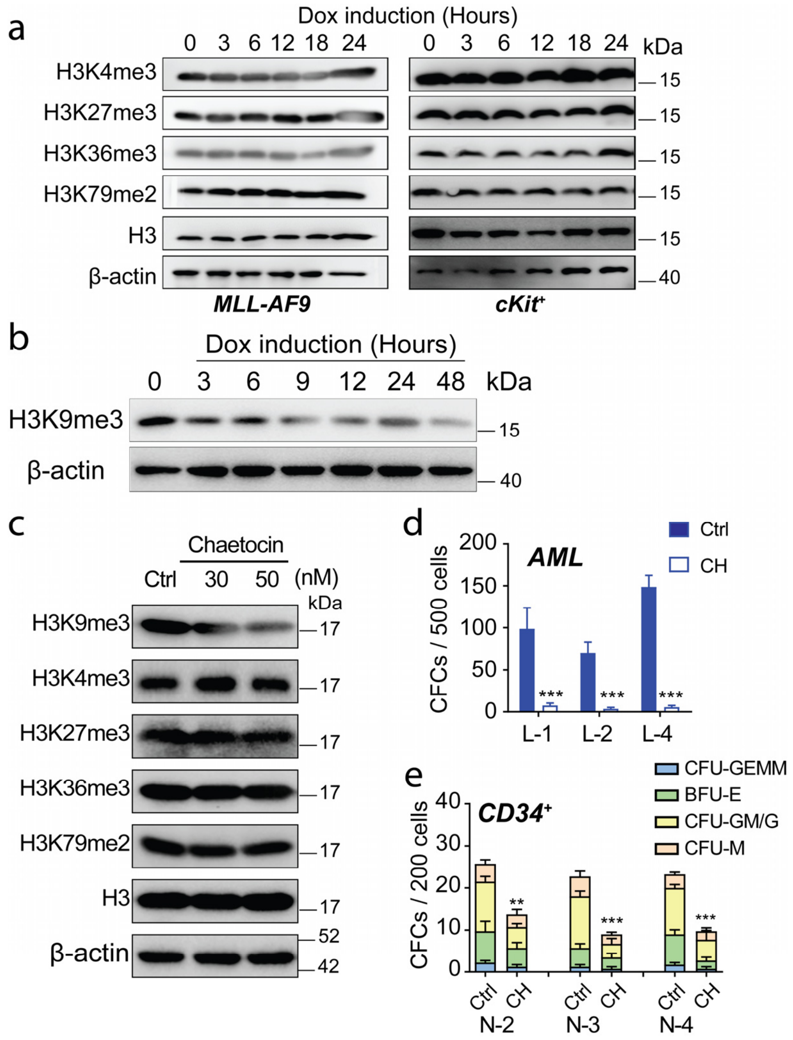

Supplementary Figure 8. Effect of chaetocin treatment on murine and human cells. (a) Western blot analysis of H3K4me3, H3K27me3, H3K36me3, H3K79me2, H3, and Actin in AML OSKM and *tet*O-OSKM cKit^{+} cells after Dox induction at different time points. Representative graphs of 3 independent experiments. (b) Western blot of H3K9me3 in AML cells transduced with *Sox2+Klf4*. The data shows the results after Dox induction at different time points. (c) Western blot analysis of H3K9me3 in murine AML cells after treatment with the indicated concentrations of chaetocin or DMSO. (d) Colony formation of human AML CD34^{+} cells after treatment with 30 nM chaetocin for 24 hr (3 independent experiments with 3 individual samples). CH, chaetocin. ***p < 0.001, two-tailed Student's t test. Error bars indicate SEM. (e) Colony formation of human normal CD34^{+} cells after treatment with 30 nM chaetocin for 24 hr (3 independent experiments with 3 individual samples).**p < 0.01, ***p < 0.001, two-tailed Student's t test. Error bars indicate SEM.

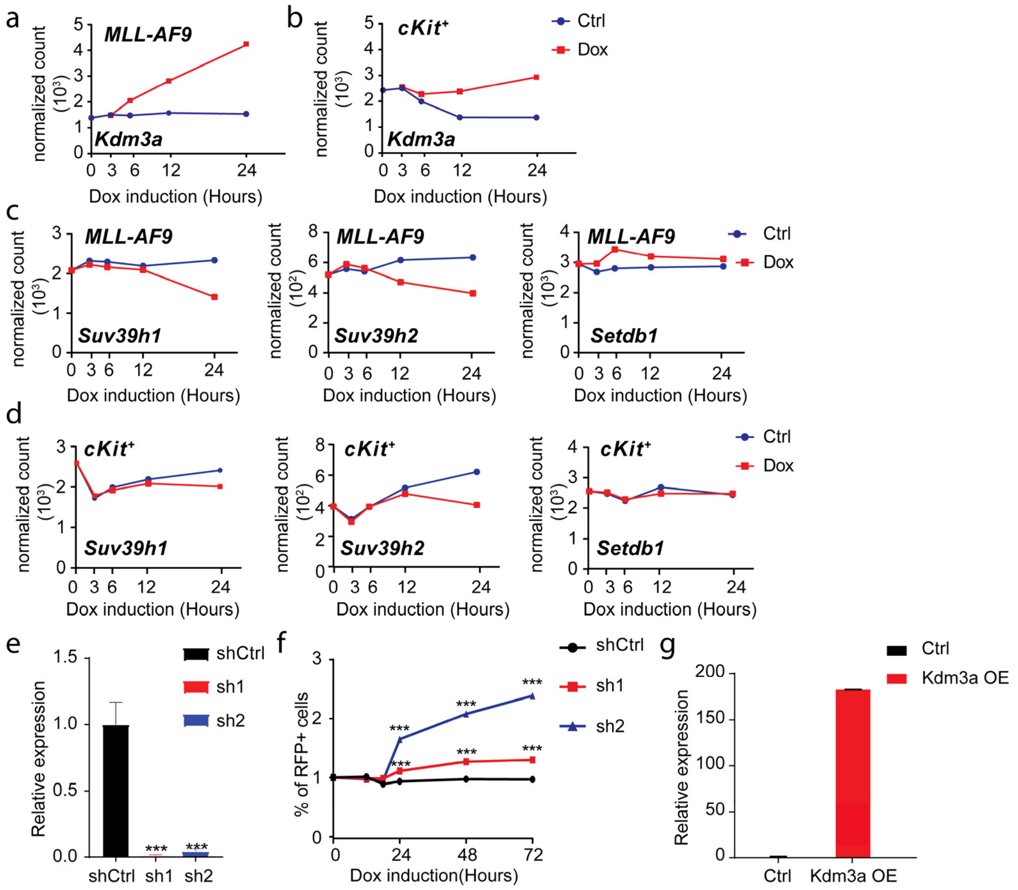

Supplementary Figure 9. The expression of H3K9 demethylase and methyltransferase. (a) RNA-seq data of *Kdm3a* expression in MLL-AF9-OSKM cells after Dox treatment. (b) RNA-seq data of *Kdm3a* expression in tetO-OSKM cKit$^+$ cells after Dox treatment. (c) RNA-seq data of *Suv39h1/h2* and *Setdb1* expression in MLL-AF9-OSKM cells after Dox treatment. (d) RNA-seq data of *Suv39h1/h2* and *Setdb1* expression in tetO-OSKM cKit$^+$ cells after Dox treatment. (e) Knockdown efficiency of *Kdm3a* shRNA in MLL-AF9-OSKM cells (n=3, 2 independent experiments). ***p < 0.001, one-way ANOVA. Error bars show SEM. (f) Relative cell counts of MLL-AF9-OSKM cells after Dox treatment. MLL-AF9-OSKM cells were transduced with LV-*Kdm3a* shRNA-RFP lentiviruses. After 48 hr culture, the transduction efficiency was analyzed by flow cytometry, and then the cells were treated with Dox. The percentage of cells expressing RFP at the starting point was normalized to 1. 2 independent experiments. ***p < 0.001, one-way ANOVA. Error bars show SEM. (g) qRT-PCR analysis of the mRNA level of *Kdm3a* gene after 48 hr of lentivirus infection in cKit$^+$ cells (n=3). Error bars show SD.

Uncropped blots related to Figure 4c

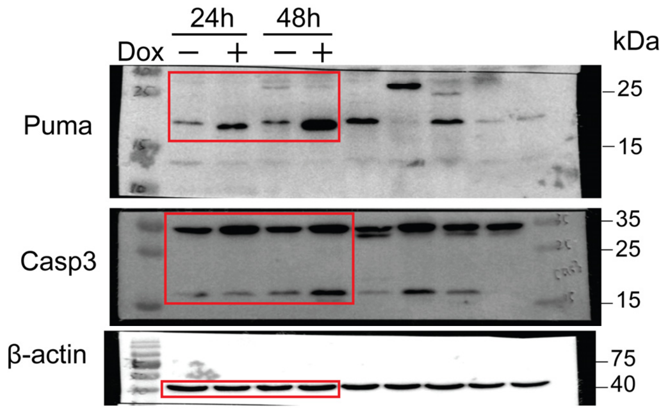

Uncropped blots related to Figure 6a

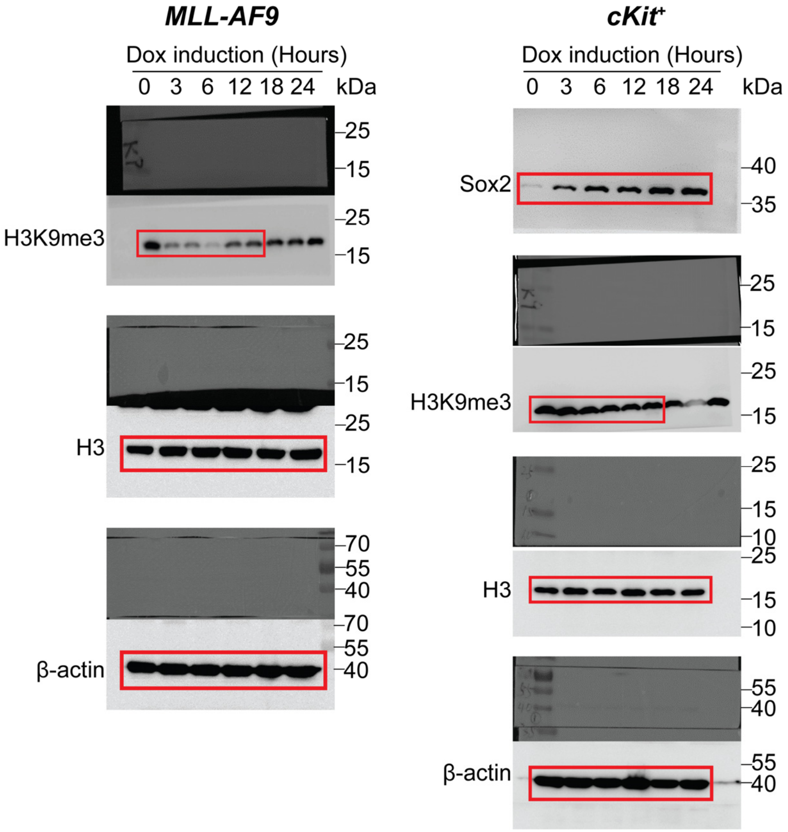

Uncropped blots related to Supplementary Fig. 8a

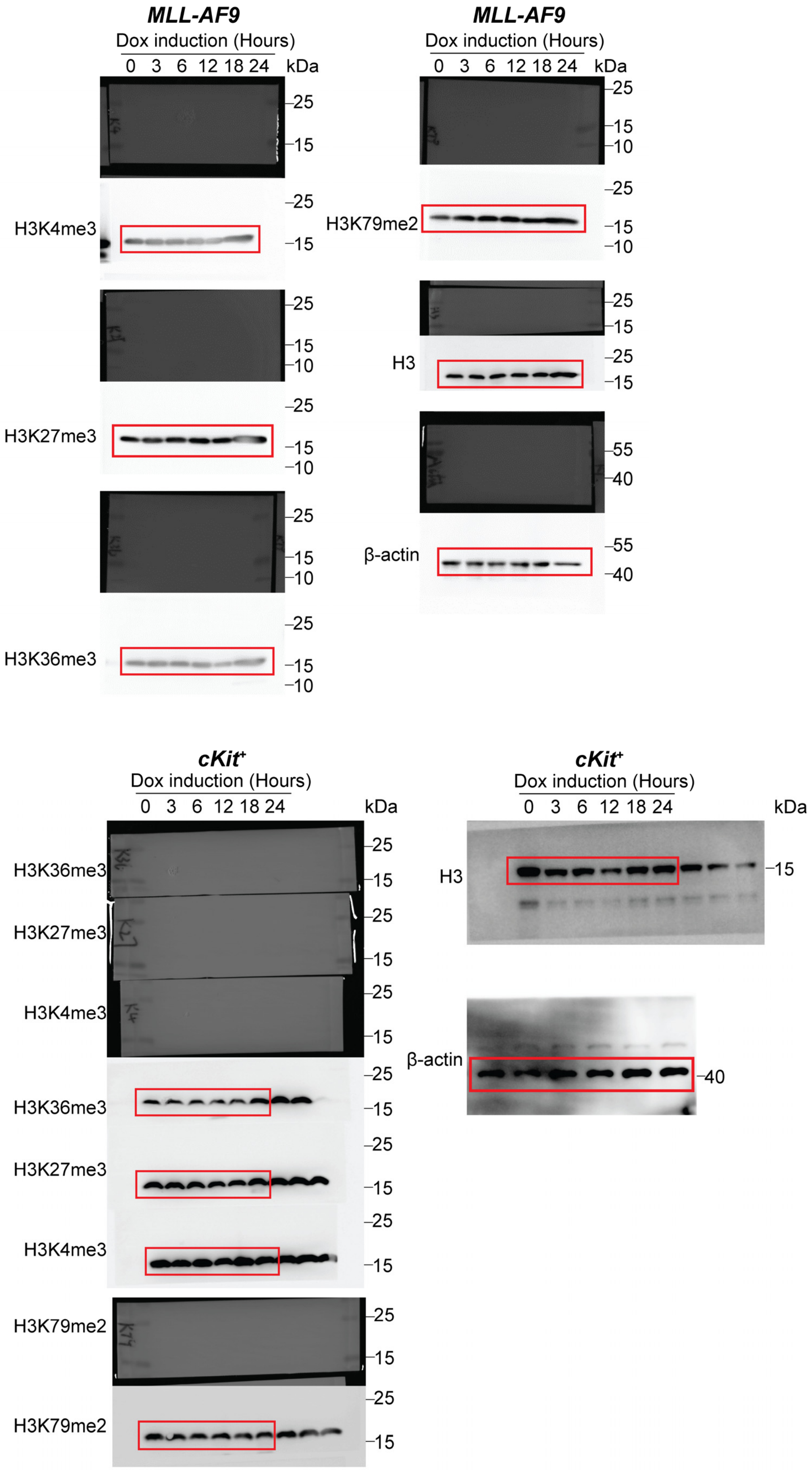

Uncropped blots related to Supplementary Fig. 8b

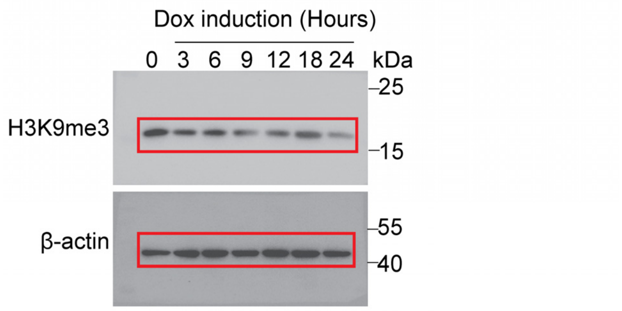

Uncropped blots related to Supplementary Fig. 8c

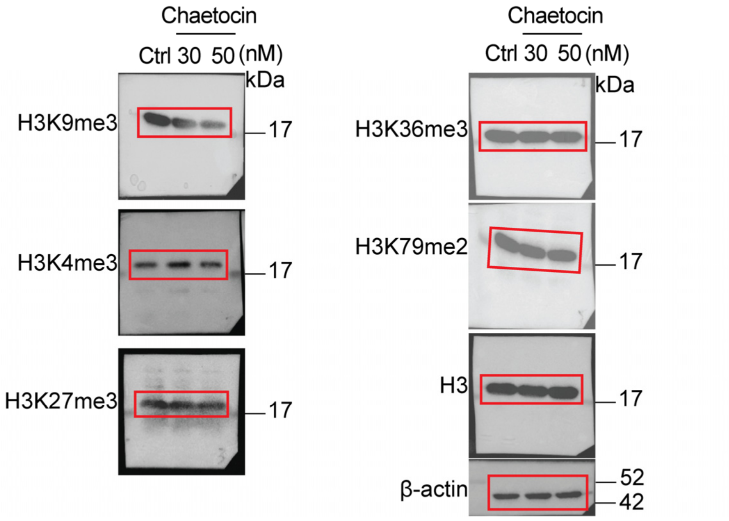

Supplementary Figure 10. Uncropped scans for Fig. 4c, Fig. 6a and supplementary Fig. 8a-8c. The relevant figures are indicated in the blot titles. The cropped areas within the red boxes are indicated.
