# Supplementary material for: Targeting of apoptosis gene loci by reprogramming factors leads to selective eradication of leukemia cells
Source: Nat Commun. 2019 Dec 6;10:5594. doi: 10.1038/s41467-019-13411-y (PMC6898631; doi:10.1038/s41467-019-13411-y)
Supplement: Supplementary file 3 — Description of Additional Supplementary Files [file 41467_2019_13411_MOESM3_ESM.pdf]

Description of Additional Supplementary Files

File Name: Supplementary Data 1

Description: ATAC-seq differential motif list
