# Supplementary material for: Targeting of apoptosis gene loci by reprogramming factors leads to selective eradication of leukemia cells
Source: Nat Commun. 2019 Dec 6;10:5594. doi: 10.1038/s41467-019-13411-y (PMC6898631; doi:10.1038/s41467-019-13411-y)
Supplement: Supplementary file 5 — Reporting Summary [file 41467_2019_13411_MOESM5_ESM.pdf]

nature research

Corresponding author(s): Kuangyu Yen, Hui Cheng and Tao Cheng

Last updated by author(s): Sep 25, 2019

Reporting Summary

Nature Research wishes to improve the reproducibility of the work that we publish. This form provides structure for consistency and transparency in reporting. For further information on Nature Research policies, see Authors & Referees and the Editorial Policy Checklist.

Statistics

For all statistical analyses, confirm that the following items are present in the figure legend, table legend, main text, or Methods section.

n/a	Confirmed	
☐	☒	The exact sample size (*n*) for each experimental group/condition, given as a discrete number and unit of measurement
☐	☒	A statement on whether measurements were taken from distinct samples or whether the same sample was measured repeatedly
☐	☒	The statistical test(s) used AND whether they are one- or two-sided<br>*Only common tests should be described solely by name; describe more complex techniques in the Methods section.*
☒	☐	A description of all covariates tested
☐	☒	A description of any assumptions or corrections, such as tests of normality and adjustment for multiple comparisons
☐	☒	A full description of the statistical parameters including central tendency (e.g. means) or other basic estimates (e.g. regression coefficient) AND variation (e.g. standard deviation) or associated estimates of uncertainty (e.g. confidence intervals)
☐	☒	For null hypothesis testing, the test statistic (e.g. *F*, *t*, *r*) with confidence intervals, effect sizes, degrees of freedom and *P* value noted<br>*Give P values as exact values whenever suitable.*
☒	☐	For Bayesian analysis, information on the choice of priors and Markov chain Monte Carlo settings
☐	☒	For hierarchical and complex designs, identification of the appropriate level for tests and full reporting of outcomes
☐	☒	Estimates of effect sizes (e.g. Cohen's *d*, Pearson's *r*), indicating how they were calculated

Our web collection on statistics for biologists contains articles on many of the points above.

Software and code

Policy information about availability of computer code

Data collection	Flow cytometry: BD FACSDive 8.<br>Microarray: Mouse Gene 2.0 ST GeneChip® arrays (Affymetrix, America).<br>RNA-seq and ATAC-seq: Illumina HiSeq XTen.
Data analysis	Microarray: Oligo package, Limma package, clusterProfiler package.<br>RNA-seq: STAR package, Samtools package, DEseq2 package, Homer package, metascape package, ComplexHeatmap.<br>ATAC-seq: BWA package, Samtools package, macs2 package, DiffBind package, Homer package, metascape package, ComplexHeatmap.<br>We used Flowjo v10 for FACS analysis and GraphPad Prism v7.0 for statistical tests.

For manuscripts utilizing custom algorithms or software that are central to the research but not yet described in published literature, software must be made available to editors/reviewers. We strongly encourage code deposition in a community repository (e.g. GitHub). See the Nature Research guidelines for submitting code & software for further information.

Data

Policy information about availability of data

All manuscripts must include a data availability statement. This statement should provide the following information, where applicable:

- Accession codes, unique identifiers, or web links for publicly available datasets
- A list of figures that have associated raw data
- A description of any restrictions on data availability

Microarray, RNA-seq and ATAC-seq datasets have been deposited in the Gene Expression Omnibus (GEO, NCBI) repository under accession number GSE64414 and GSE121123

Field-specific reporting

Please select the one below that is the best fit for your research. If you are not sure, read the appropriate sections before making your selection.

☒ Life sciences ☐ Behavioural & social sciences ☐ Ecological, evolutionary & environmental sciences

For a reference copy of the document with all sections, see nature.com/documents/nr-reporting-summary-flat.pdf

Life sciences study design

All studies must disclose on these points even when the disclosure is negative.

Sample size	No statistical methods were used to predetermine the experimental sample size.
Data exclusions	No data were excluded.
Replication	For all figures, multiple independent experiments were performed and all attempts at replicating observation as described in the manuscript were successful.
Randomization	Animals and the in vitro experimental samples were allocated randomly
Blinding	Blinding was not performed.

Reporting for specific materials, systems and methods

We require information from authors about some types of materials, experimental systems and methods used in many studies. Here, indicate whether each material, system or method listed is relevant to your study. If you are not sure if a list item applies to your research, read the appropriate section before selecting a response.

Materials & experimental systems

n/a	Involved in the study	
☐	☒	Antibodies
☐	☒	Eukaryotic cell lines
☒	☐	Palaeontology
☐	☒	Animals and other organisms
☐	☒	Human research participants
☒	☐	Clinical data

Methods

n/a	Involved in the study	
☒	☐	ChIP-seq
☐	☒	Flow cytometry
☒	☐	MRI-based neuroimaging

Antibodies

Antibodies used

Antibodies used in flow cytometry:
PerCP-Cy5.5 CD45.1 (eBioscience, Clone: A20, Cat: 45-0453-82, Dilution: 1:200)
FITC CD45.2 (eBioscience, Clone:104, Cat: 11-0454-82, Dilution: 1:200)
PE-Cy7 Sca-1 (eBioscience, Clone:D7, Cat: 25-5981-82, Dilution: 1:200)
APC c-Kit (eBioscience, Clone:2B8, Cat: 17-1171-82, Dilution: 1:200)
APC-Cy7 CD3 (Biolegend, Clone:145-2C11, Cat: 100330, Dilution: 1:400)
APC-Cy7 CD4 (Biolegend, Clone: GK1.5, Cat: 100414, Dilution: 1:400)
APC-Cy7 CD8 (Biolegend, Clone: 53-6.7, Cat: 100714, Dilution: 1:400)
APC-Cy7 B220 (Biolegend, Clone: RA3-6B2, Cat: 103224, Dilution: 1:400)
APC-Cy7 Gr-1 (Biolegend, Clone: RB6-8C5, Cat: 108424, Dilution: 1:400)
APC-Cy7 Mac-1 (Biolegend, Clone: M1/70, Cat: 101226, Dilution: 1:400)
APC-Cy7 Ter119 (Biolegend, Clone: TER-119, Cat: 116223, Dilution: 1:400)
APC-Cy7 CD127 (Biolegend, Clone: A7R34, Cat: 135040, Dilution: 1:400)
APC Annexin V (BD Bioscience, Cat: 550475, Dilution: 1:100)

Antibodies used in Western blotting:
p53 (Cell Signaling, Cat: 2527S, Dilution: 1:500)
Puma (Abcam, Cat: ab9643, Dilution: 1:500)
caspase-3 (Cell Signaling, Cat: 9662S, Dilution: 1:1,000)
β-actin (Cell Signaling, Cat: 3700S, Dilution 1:5,000)
H3 (Abcam, Cat: ab1791, Dilution 1:5,000)
H3K9me3 (Abcam, Cat: ab8898, Dilution 1:1,000)
Sox2 (Abcam, Cat: ab93689, Dilution 1:1,000)
H3K4me3 (Abcam, Cat: ab8580, Dilution: 1:1,000)
H3K27me3 (Abcam, Cat: ab6002, Dilution: 1:1,000)

H3K79me2 (Abcam, Cat: ab3594, Dilution: 1:1,000)
H3K36me3 (Abcam, Cat: ab194677, Dilution: 1:1,000)

Validation	All antibodies used in this work were purchased from companies, and validated by the manufacturers and by extensive use in published work.

Eukaryotic cell lines

Policy information about cell lines

Cell line source(s)	293T and THP-1 cells lines were obtained from SKLEH's experimental pathology cell bank.
Authentication	293T and THP-1 cells were authenticated by examination of morphology and growth characteristics.
Mycoplasma contamination	Cells were confirmed to be mycoplasma free.
Commonly misidentified lines (See ICLAC register)	No commonly misidentified lines were used in this study.

Animals and other organisms

Policy information about studies involving animals; ARRIVE guidelines recommended for reporting animal research

Laboratory animals	B6-Ly5.1, B6-Ly5.2 and NOD/SCID mice were purchased from the animal facility of the State Key Laboratory of Experimental Hematology (SKLEH, Tianjin, China). The OSKM mice were gifts from Dr. Shaorong Gao (Tongji University, Shanghai, China). All animal procedures complied with the animal care guidelines approved by the Institutional Animal Care and Use Committees of the SKLEH and and the Institute of Hematology.
Wild animals	The study did not involve wild animals.
Field-collected samples	The study did not involve field-collected samples.
Ethics oversight	All animal procedures complied with the animal care guidelines approved by the Institutional Animal Care and Use Committees of the SKLEH and the Institute of Hematology.

Note that full information on the approval of the study protocol must also be provided in the manuscript.

Human research participants

Policy information about studies involving human research participants

Population characteristics	Normal cord blood mononuclear cells were obtained from the Tianjin Central Hospital of Gynecology and Obstetrics. Primary human AML blasts were obtained from SKLEH's experimental pathology cell bank. According to the regulations of the institutional ethics review boards from the Institute of Hematology and Blood Diseases Hospital, Chinese Academy of Medical Sciences and Peking Union Medical College, informed consent was signed by all patients.
Recruitment	Primary human AML blasts were obtained from SKLEH's experimental pathology cell bank,
Ethics oversight	According to the regulations of the institutional ethics review boards from the Institute of Hematology and Blood Diseases Hospital, Chinese Academy of Medical Sciences and Peking Union Medical College, informed consent was signed by all patients.

Note that full information on the approval of the study protocol must also be provided in the manuscript.

Flow Cytometry

Plots

Confirm that:

- [x] The axis labels state the marker and fluorochrome used (e.g. CD4-FITC).
- [x] The axis scales are clearly visible. Include numbers along axes only for bottom left plot of group (a 'group' is an analysis of identical markers).
- [x] All plots are contour plots with outliers or pseudocolor plots.
- [x] A numerical value for number of cells or percentage (with statistics) is provided.

Methodology

Sample preparation	For cell sorting experiments using mouse HSPCs, cKit+ cells were enriched before flow cytometry using cKit magnetic beads (Miltenyi Biotec). Subsequently, the cells were stained with a lineage cocktail (Gr-1, Mac-1, B220, CD3, CD4, CD8 and Ter-119) and with cKit and Sca-1 antibodies. DAPI (1 mg/mL; Sigma-Aldrich) was used to exclude dead cells at all times. For LSC analysis,

nucleated bone marrow cells were stained with lineage-specific antibodies (CD3, CD4, CD8, B220, Gr-1, Ter119 and CD127) and with Sca-1 and cKit antibodies. For apoptotic analysis, the cells were stained with Annexin V and 7-AAD according to the manufacturer's recommendations (BD Biosciences).

Instrument	A modified LSR II flow cytometer with four lasers (355 nm, 488 nm, 561 nm, and 633 nm) was used for analyzing, and an Aria III flow cytometer with four lasers (375 nm, 488 nm, 561 nm, and 633 nm) was used for sorting
Software	The analyses were performed using FACSDiVa and FlowJo (Tree Star) software.
Cell population abundance	FACS machine cell sorting efficiency was confirmed by flow cytometric analysis of post-sorted cells.
Gating strategy	FSC-A/SSC-A for mononuclear cells, FSC-H/FSC-W followed by SSC-H/SSC-W for singlets. For figure 1, GFP+ cells were gated. For Figure 2, DAPI- was gated, followed by CD45.1 and CD45.2 gate. For Figure 3, GFP+ cells were gated, followed by Annevin V and 7-AAD gate. For Figure 5, RFP cells were gated, followed by Annevin V and DAPI gate. Cells were gated based on positive and negative markers.

☒ Tick this box to confirm that a figure exemplifying the gating strategy is provided in the Supplementary Information.
